# A new approach toward the total synthesis of (+)-batzellaside B

**DOI:** 10.3762/bjoc.8.210

**Published:** 2012-10-25

**Authors:** Jolanta Wierzejska, Shin-ichi Motogoe, Yuto Makino, Tetsuya Sengoku, Masaki Takahashi, Hidemi Yoda

**Affiliations:** 1 Department of Materials Science, Faculty of Engineering, Shizuoka University, 3-5-1 Johoku, Naka-ku, Hamamatsu, Shizuoka 432-8561, Japan

**Keywords:** asymmetric dihydroxylation, (+)-batzellaside B, iminosugar, L-pyroglutamic acid, total synthesis

## Abstract

A new synthetic approach to (+)-batzellaside B from naturally abundant L-pyroglutamic acid is presented in this article. The key synthetic step involves Sharpless asymmetric dihydroxylation of an olefinic substrate functionalized with an acetoxy group to introduce two chiral centres diastereoselectively into the structure. Heterocyclic hemiaminal **4**, which could be converted from the resulting product, was found to provide stereospecific access to enantiomerically enriched allylated intermediate, offering better prospects for the total synthesis of this natural product.

## Introduction

**Figure 1:**
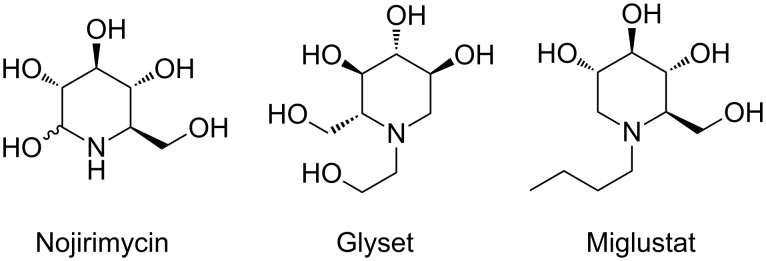
Examples of naturally occurring iminosugars.

Iminosugars, monosaccharide analogues in which the endocyclic oxygen has been replaced by nitrogen, display beneficial therapeutic activity as sugar-mimicking glycosidase inhibitors [1–4]. Since the discovery of nojirimycin (Figure 1), which was isolated from *Streptomyces roseochromogenes* R-468 and *S. lavendulae* SF-425 in the 1960s [5], this class of compounds has attracted a great deal of interest in the medical community due to their promising pharmaceutical potential as antidiabetic [6], antitumor [7] and antiviral [8] agents. Undoubtedly, approval of Glyset [9] and Miglustat [10] (Figure 1) for treatment of type II diabetes and Gaucher disease, respectively, has imparted therapeutic applications of this class of natural products.

**Figure 2:**
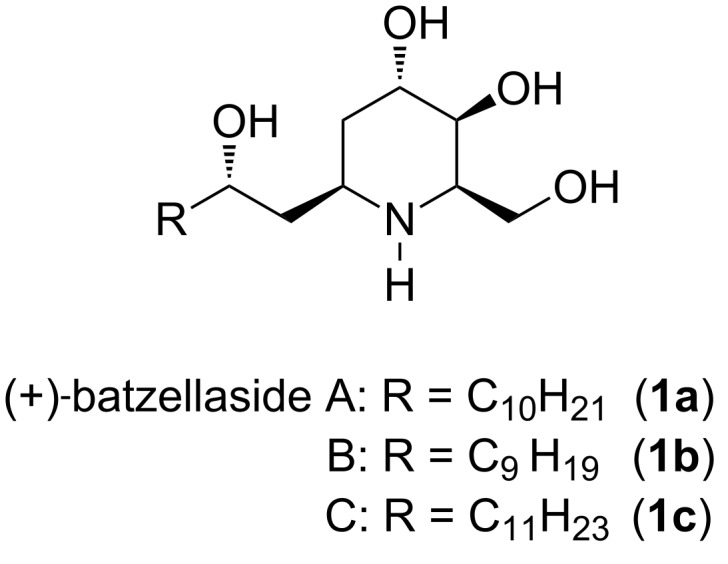
The chemical structures of (+)-batzellasides A–C (**1a**–**c**).

In this context, (+)-batzellasides A–C (**1a**–**c**) (Figure 2), *C*-alkylated azasugars isolated in 2004 from a sponge *Batzella* sp. collected off the west coast of Madagascar, represent the first example of iminosugars from a marine organism [11]. These naturally occurring products have been demonstrated to retain a remarkably high degree of potency against *Staphylococcus epidermidis* with MICs of ≤6.3 μg/mL, thus serving as new potent antibacterial agents [11]. As a part of our research program on the synthesis and investigation of biologically active natural products [12–18], we have pursued the synthetic elaboration of (+)-batzellaside B (**1b**) as a represent member of this new class of iminosugar alkaloids.

**Scheme 1:**
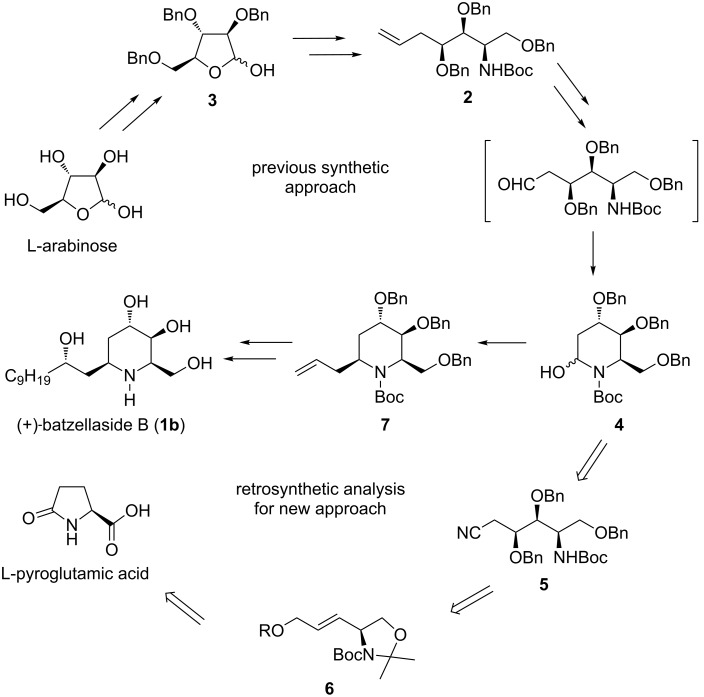
Our previous approach to (+)-batzellaside B and retrosynthetic analysis for the new synthetic strategy.

In a previous publication, we communicated the first total synthesis of (+)-batzellaside B (**1b**) by the use of L-arabinose, wherein the absolute stereochemistry of this natural product was completely established by the modified Mosher analysis of a synthetic intermediate prepared through a separate route (Scheme 1) [19]. In this approach involving nucleophilic cyclization of acyclic aldehyde in situ generated from olefin **2** for constructing the piperidine ring system, proper synthetic manipulation of the three inherent stereocenters contained in the chiral source was a key strategy for ensuring effective stereocontrol to achieve completion of the target-directed synthesis. The overall yield in 22 steps from 2,3,5-tri-*O*-benzyl-L-arabinose (**3**) was 3.9%. Although **1b** and its related analogues are now accessible through the pathway established above, pursuing a new synthetic approach strongly appealed to us, because the existing route is still rather unsatisfactory mainly in terms of the insufficient supply and the time-consuming preparation of the tribenzyl ether **3**, which is not commercially available [20]. We thus decided to explore new alternative strategies allowing for a more efficient and convenient access to this natural product and its derivatives. From a retrosynthetic point of view, L-pyroglutamic acid, whose rich natural abundance makes it a commercially and economically viable substrate [21], can be envisaged as a potentially practical starting material for this purpose (Scheme 1). Furthermore, it can be considered that the heterocyclic hemiaminal **4**, a common precursor of the target molecule for both synthetic strategies, would be derived by an analogous cyclization of the acyclic aldehyde generated in situ from cyanide **5**. In this proposed strategy, the key transformation will involve Sharpless asymmetric dihydroxylation to install stereoselectively the hydroxy groups at C3 and C4 positions of the olefinic substrate **6**, and an intramolecular cyclization of aldehyde generated in situ from **5** to construct the piperidine ring system.

The present publication describes the results of our continuing challenges associated with this targeted natural product on the basis of the above synthetic strategy. As a result, and in addition to improvement of the inefficient stereoselectivity observed in allylation process from **4** to **7**, we established the new alternative synthetic route to (+)-batzellaside B from L-pyroglutamic acid, which offers the advantages of convenience and simplicity of total synthesis.

## Results and Discussion

**Scheme 2:**
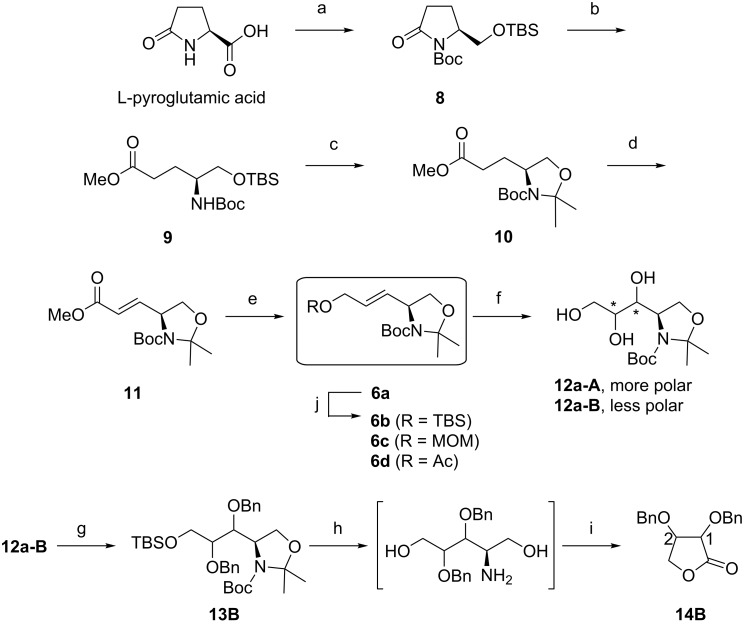
Reagents and conditions: (a) see [22]; (b) MeONa, MeOH, rt; 98%; (c) (i) *p*-TsOH, MeOH, rt; (ii) BF_3_·Et_2_O, 2,2-DMP, acetone, rt; 93% (two steps); (d) (i) LDA, HMPA, PhSeBr, THF, −78 °C; (ii) *m*-CPBA, CH_2_Cl_2_, −40 °C; 90% (two steps); (e) DIBAL-H, THF, 0 °C; 95%; (f) OsO_4_, NMO, *t*-BuOH/H_2_O (1/1), rt; 30%, **12a-A**/**12a-B** = 24/76; (g) (i) TBSCl, Et_3_N, CH_2_Cl_2_, rt; (ii) BnBr, NaH, Bu_4_NI, THF, rt; (h) (i) *p*-TsOH, MeOH, rt; (ii) TFA, CH_2_Cl_2_, rt; (i) (i) NaIO_4_, Et_2_O/H_2_O (1/1), rt; (ii) PCC, MS 4 Å, CH_2_Cl_2_, rt; 17% (six steps); (j) TBSCl, imidazole, DMF; 77% (**6b**); or MOMCl, NaH, THF; 68% (**6c**); or Ac_2_O, DMAP, Et_3_N, CH_2_Cl_2_; 99% (**6d**).

The synthesis began with the preparation of *N*-Boc-protected γ-lactam **8** by stepwise functionalization of L-pyroglutamic acid, using literature procedures (Scheme 2) [22]. As indicated previously, this compound underwent efficient ring opening upon treatment with sodium methoxide in methanol to provide acyclic ester **9** in 98% yield [23]. In the next step, the TBS protecting group in **9** was removed by exposure to methanolic *p*-TsOH to give the corresponding alcohol, which was then subjected to reaction with 2,2-dimethoxypropane (2,2-DMP) in the presence of BF_3_·Et_2_O [24] to produce *N*,*O*-acetonide **10** in 93% yield over two steps. For the preparation of the *E*-isomer of α,β-unsaturated ester **11**, Wittig–Horner reaction employing Garner’s aldehyde has been well known [25–26]; however, we selected stereoselective olefination through deprotonation of **10** with LDA followed by the addition of phenylselenyl bromide [27] and subsequent oxidative elimination of the resulting phenylseleno group with *m*-CPBA according to our previous report [28], which gave *E*-isomer **11** in quantitative geometric purity and 90% yield over two steps. Then, the ester moiety of this compound was reduced with DIBAL-H to the corresponding hydroxymethyl functionality to afford **6a** in 95% yield [29].

With this olefinic compound in hand, we examined the dihydroxylation of **6a**, which was carried out with OsO_4_ and NMO in 50% aqueous *t*-BuOH at room temperature [30–33]. Under these conditions, **6a** provided an approximately 1:3 ratio of the more- and less-polar diastereomeric triols **12a-A** and **12a-B**, respectively, in 30% yield. These diastereomers were separated by silica-gel column chromatography and then derivatized to *trans*-1,2-dibenzyloxy-substituted γ-lactone, which allows a precise assignment of the absolute configuration by comparison with the specific optical rotation value [34]. Accordingly, the primary hydroxy group of **12a-B** was chemoselectively protected as the TBS ether [35] and the remaining diol moiety was then etherified with NaH and benzyl bromide, affording **13B** in a crude form. Deprotection of the acetal and TBS groups of this product was carried out by stepwise reactions with *p*-TsOH and TFA to give a dihydroxyamine intermediate, which underwent spontaneous cyclization upon treatment with NaIO_4_ [36] followed by PCC oxidation to form the corresponding *trans*-1,2-dibenzyloxy-substituted γ-lactone **14B** in 17% yield over six steps. For comparison, optical rotation measurement was performed on a solution of **14B** in CHCl_3_ at *c* 0.36. Indeed, this compound exhibited optical activity with an [α]_D_^24^ value of −51.6, indicative of an opposite sense of absolute configuration in comparison to the literature data given for (1*S*,2*S*)-dibenzyloxy γ-lactone ([α]_D_^25^ +60.1, *c* 1.0, CHCl_3_) [34]. From this, we can conclude, as seen in Scheme 2, that the more polar triol **12a-A** obtained as a minor component of this dihydroxylation process could be identified as the (1*S*,2*S*)-isomer that should be supplied to advance our ongoing synthetic strategy, albeit with low yield for its preparation.

In an effort to improve selectivity of stereogenesis for the dihydroxylation step, we prepared three other olefinic substrates, in which the hydroxy group in **6a** was replaced by *tert*-butyldimethylsilyloxy (TBSO), methoxymethyloxy (MOMO) and acetoxy (AcO) groups, according to standard procedures [37–38], to give moderate to high yields (68–99%) of **6b**–**d**, respectively. Having the four different olefinic compounds **6a**–**d** available, we turned to an asymmetric technique of dihydroxylation to synthesize the (1*S*,2*S*)-constituent in preference to another (Table 1). Indeed, the Sharpless methodology was initially applied to **6a**, by carrying out the reactions at room temperature under a standard set of the asymmetric hydroxylation conditions [39–41]. Using AD-mix-α and -β, we obtained mixtures of **12a-A** and **12a-B** in 45:55 and 13:87 ratios with overall isolated yields of 32 and 77%, respectively (Table 1, entries 1 and 2). Analogously, the asymmetric dihydroxylations of **6b** and **6c** produced predominantly the undesired less-polar diastereomers **12b-B** and **12c-B**, which could be converted by acidic deprotection to **12a-B** (Table 1, entries 3,4 and 6), while the reaction of **6c** with AD-mix-α afforded a 50:50 mixture of diastereomers (Table 1, entry 5).

**Table 1:** Asymmetric dihydroxylation of **6a**–**d**.

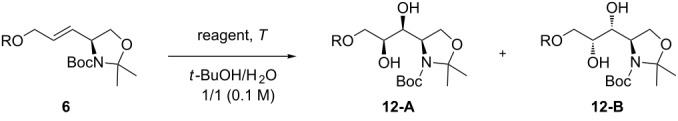					

Entry	**6**	R	Reagent (amount [mol %])	*T*	Yield [%]^a^ (**12-A**/**12-B**)^b^

1	**a**	H	AD-mix-α (0.5), MeSO_2_NH_2_ (100)	rt	32 (45/55)
2	**a**	H	AD-mix-β (0.5), MeSO_2_NH_2_ (100)	rt	77 (13/87)
3	**b**	TBS	AD-mix-α (0.5), MeSO_2_NH_2_ (100)	rt	33 (14/86)
4	**b**	TBS	AD-mix-β (0.5), MeSO_2_NH_2_ (100)	rt	35 (40/60)
5	**c**	MOM	AD-mix-α (0.5), MeSO_2_NH_2_ (100)	rt	52 (50/50)
6	**c**	MOM	AD-mix-β (0.5), MeSO_2_NH_2_ (100)	rt	88 (0/100)
7	**d**	Ac	AD-mix-α (0.5), MeSO_2_NH_2_ (100)	rt	48 (69/31)
8	**d**	Ac	AD-mix-β (0.5), MeSO_2_NH_2_ (100)	rt	51 (9/91)
9	**d**	Ac	AD-mix-α (0.5), MeSO_2_NH_2_ (100)	0 °C	54 (78/22)
10	**d**	Ac	AD-mix-α (0.5)	0 °C	52 (84/16)
11	**d**	Ac	AD-mix-α (0.5), (DHQ)_2_PHAL (10)	0 °C	53 (83/17)

^a^Isolated yield. ^b^Diastereomeric ratios were determined by ^1^H NMR (300 MHz).

A remarkable change in the product profile occurred when **6d** was used for this reaction. By employing AD-mix-α under the above reaction conditions, **6d** gave rise to a 69:31 mixture of **12d-A** and **12d-B** in 48% yield, leading to **12a-A** and **12a-B** through hydrolysis under basic conditions, respectively, whereas the use of AD-mix-β resulted in predominant formation of the undesired diastereomer (Table 1, entries 7 and 8). The above observations led us to explore the AD-mix-α-mediated reaction of **6d** at lower temperatures. When the reaction was performed at 0 °C with the same set of the reagents, the product selectivity for **12d-A** was slightly improved (Table 1, entry 9). Remarkably, **6d** underwent slow reaction in the absence of MeSO_2_NH_2_ to give the product mixture in 52% yield, and the diastereomeric ratio could be further enriched to 84:16 (Table 1, entry 10). Additionally, a similar result was obtained by carrying out the reaction using 0.1 equiv of (DHQ)_2_PHAL (53%, 83:17 in Table 1, entry 11). The above observations clearly suggested a possibility of improving the product selectivity by lowering the reaction temperature and/or introducing an additional catalytic amount of chiral ligand.

**Scheme 3:**
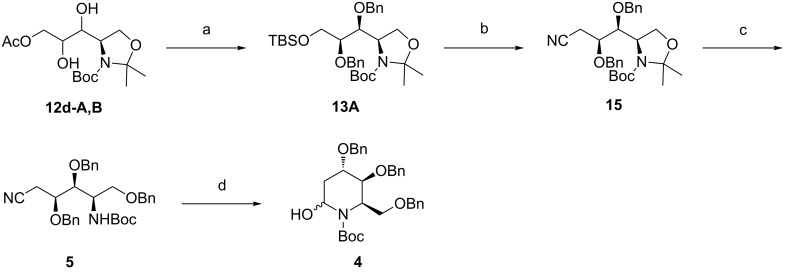
Reagents and conditions: (a) (i) K_2_CO_3_, MeOH, rt; (ii) TBSCl, Et_3_N, CH_2_Cl_2_, rt; (iii) BnBr, NaH, Bu_4_NI, THF, rt; 50% (three steps); (b) (i) TBAF, THF, rt; (ii) TsCl, pyridine, 0 °C to rt; (iii) NaCN, NaHCO_3,_ DMSO, 60 °C; 80% (three steps); (c) (i) *p*-TsOH, MeOH, rt; (ii) BnBr, Ag_2_O, AcOEt, rt; 67% (two steps); (d) DIBAL-H, toluene, −78 °C; 67%.

Having established the optimized conditions for the preparation of **12d-A**, our next objective was the construction of the piperidine ring system. As shown in Scheme 3, the acetyl group of the diastereomerically enriched mixture of **12d-A** and **12d-B** was removed by exposure to K_2_CO_3_ in methanol to give the separable mixture of alcohols **12a-A** and **12a-B**, respectively [42]. After purification by silica-gel column chromatography, **12a-A** was subjected to the TBS protection–benzylation sequence, as illustrated for the preparation of **13B**, to generate **13A** in 50% yield over three steps. In the next step, deprotection of the TBS group with TBAF [37] and subsequent tosylation of the resulting hydroxy group with TsCl in the presence of pyridine was carried out to yield the corresponding tosylate, whose activated ester group could be displaced with NaCN in DMSO to provide cyanide **15** in 80% yield over three steps [43]. Then, the *N*,*O*-acetonide group of **15** was cleaved upon treatment with *p*-TsOH in methanol [44], and the released primary hydroxy group was subsequently protected as the benzyl ether to produce the key intermediate **5** in 67% yield over two steps. As expected, conversion of this compound into the heterocyclic hemiaminal **4** was achieved in one pot with DIBAL-H by the formation of the aldehyde followed by spontaneous intramolecular cyclization with a yield of 67%. The structural identity of this product was precisely confirmed by ^1^H NMR spectroscopic data, which proved to be in good agreement with those on record for **4** [19]. Hence, we can conclude that a formal total synthesis of (+)-batzellasides B was accomplished, considering that the synthetic route from **4** to **1b** has been established previously.

At this point, it was thus suggested that the remaining challenge was to improve the stereoselectivity of the allylation of **4**. In the first approach of our previous work [19], we demonstrated that the heterocyclic hemiaminal **4** was allylated by following a protocol using allyltributylstannane (AllylSnBu_3_) and *tert*-butyldimethylsilyl triflate (TBSOTf) at −78 °C in toluene to furnish the product **7** as a 69:31 mixture of diastereomeric isomers in 96% yield (Table 2, entry 1). Despite the appealing performance observed in the above synthetic process, this reaction protocol has the major disadvantage of low stereoselectivity causing operational inconvenience associated with the laborious chromatographic separation of the two stereoisomers. From the practical considerations, we next explored a more efficient synthetic method for the stereoselective allylation of **4** using an appropriate combination of allylic reagent and Lewis acid to produce the desired diastereomer **7** preferentially. Beginning with a reaction employing indium chloride (InCl_3_) instead of TBSOTf at 0 °C in dichloromethane, we observed no substantial improvement in the stereoselectivity of the allylation, affording a 44:56 mixture of **7** and **7’** in quantitative yield (Table 2, entry 2). A much greater preference for the formation of **7** was observed in the cases where allyltrimethylsilane (AllylTMS) was used as an allylic reagent (Table 2, entries 3–5). In fact, the reaction carried out with zinc chloride (ZnCl_2_) at room temperature in toluene led to exclusive stereoselectivity for **7** with 98% de, albeit in low yield (24%, Table 2, entry 3). Furthermore, the use of TBSOTf as a Lewis acid resulted in significant enhancement of the reaction rate to give almost the same stereochemical outcome (98 and 92% de) with slightly and moderately higher yields (29 and 41%) for the periods of 2 and 3 h (Table 2, entries 4 and 5), respectively. In spite of its increased susceptibility, which should be discriminated from those of unsubstituted structural systems [45–50], it became apparent that this multiply functionalized hemiaminal **4** is well tolerated to undergo direct allylation with the silyl reagents. The results of the above investigations provide one particularly successful route that has the potential to allow direct asymmetric access to the advanced-stage intermediate **7** under precise stereochemical control as well as for circumventing the purification problems related to the diastereomeric impurity in this product.

**Table 2:** Diastereoselective allylation of **4**.

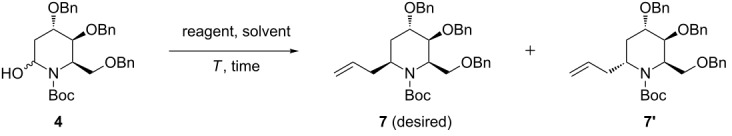					

Entry	Reagent (amount [equiv])	Solvent	*T*	Time	Yield [%]^a^ (**7**/**7’**)^b^

1	AllylSnBu_3_ (3.0), TBSOTf (1.5)	toluene	−78 °C	9 h	96 (69/31)
2	AllylSnBu_3_ (3.0), InCl_3_ (1.5)	CH_2_Cl_2_	0 °C	0.75 h	quant. (44/56)
3	AllylTMS (4.0), ZnCl_2_ (4.0)	toluene	rt	16 h	24 (99/1)
4	AllylTMS (4.0), TBSOTf (2.0)	CH_2_Cl_2_	−78 to −45 °C	2 h	29 (99/1)
5	AllylTMS (10.0), TBSOTf (1.5)	toluene	−78 °C	3 h	41 (96/4)

^a^Isolated yield. ^b^Diastereomeric ratios were determined by ^1^H NMR (300 MHz).

## Conclusion

We have described a new synthetic approach to (+)-batzellaside B from L-pyroglutamic acid. Starting from this chiral material, the formal total synthesis of the heterocyclic hemiaminal **4**, a key intermediate elaborated commonly in the first total synthesis, has been achieved in an efficient 21-step protocol in 7.1% overall yield. Furthermore, the stereospecificity in the allylation of **4** has been exemplified by performing the procedures with AllylTMS and two types of Lewis acids, which allows for simpler synthetic operation due to the ease of purification of the products. The present study clearly demonstrates that L-pyroglutamic acid can be used as a versatile chiral source for synthesizing this class of biologically potent piperidine alkaloids and related analogues.

## Supporting Information

File 1 Full experimental details and characterization data.

## References

[1] Ganem B (1996). Acc Chem Res.

[2] Stutz A E (1999). Iminosugars as Glycosidase Inhibitors: Norjirimycin and Beyond.

[3] Felpin F-X, Lebreton J (2003). Eur J Org Chem.

[4] Afarinkia K, Bahar A (2005). Tetrahedron: Asymmetry.

[5] Inoue S, Tsuruoka T, Niida T (1966). J Antibiot.

[6] Somsak L, Nagya V, Hadady Z, Docsa T, Gergely P (2003). Curr Pharm Des.

[7] Greimel P, Spreitz J, Stutz A E, Wrodnigg T M (2003). Curr Top Med Chem.

[8] Nishimura Y (2003). Curr Top Med Chem.

[9] Jacob G S (1995). Curr Opin Struct Biol.

[10] McCormack P L, Goa K L (2003). Drugs.

[11] Segraves N L, Crews P (2005). J Nat Prod.

[12] Sengoku T, Murata Y, Mitamura H, Takahashi M, Yoda H (2012). Tetrahedron Lett.

[13] Sengoku T, Wierzejska J, Takahashi M, Yoda H (2010). Synlett.

[14] Takahashi M, Suzuki T, Wierzejska J, Sengoku T, Yoda H (2010). Tetrahedron Lett.

[15] Sengoku T, Satoh Y, Takahashi M, Yoda H (2009). Tetrahedron Lett.

[16] Takahashi M, Maehara T, Sengoku T, Fujita N, Takabe K, Yoda H (2008). Tetrahedron.

[17] Matsuura D, Mitsui T, Sengoku T, Takahashi M, Yoda H (2008). Tetrahedron.

[18] Takahashi M, Takada K, Matsuura D, Takabe K, Yoda H (2007). Heterocycles.

[19] Wierzejska J, Ohshima M, Inuzuka T, Sengoku T, Takahashi M, Yoda H (2011). Tetrahedron Lett.

[20] Tejima S, Fletcher H G (1963). J Org Chem.

[21] Panday S K, Prasad J, Dikshit D K (2009). Tetrahedron: Asymmetry.

[22] Sengoku T, Satoh Y, Oshima M, Takahashi M, Yoda H (2008). Tetrahedron.

[23] Flynn D L, Zelle R E, Grieco P A (1983). J Org Chem.

[24] Xu Z, Zhang F, Zhang L, Jia Y (2011). Org Biomol Chem.

[25] Jako I, Uiber P, Mann A, Wermuth C-G, Boulanger T, Norberg B, Evrard G, Durant F (1991). J Org Chem.

[26] Jako I, Uiber P, Mann A, Taddei M, Wermuth C-G (1990). Tetrahedron Lett.

[27] Silverman R B, Invergo B J, Mathew J (1986). J Med Chem.

[28] Yoda H, Shirai T, Katagiri T, Takabe K, Kimata K, Hosoya K (1990). Chem Lett.

[29] Spangenberg T, Schoenfelder A, Breit B, Mann A (2010). Eur J Org Chem.

[30] Dondoni A, Merino P, Perrone D (1993). Tetrahedron.

[31] Krishna P R, Reddy P S (2009). Synlett.

[32] Passiniemi M, Koskinen A M P (2010). Synthesis.

[33] Upadhyay P K, Kumar P (2010). Synthesis.

[34] Pabba J, Rempel B P, Withers S G, Vasella A (2006). Helv Chim Acta.

[35] Reetz M T, Kesseler K (1985). J Org Chem.

[36] House H O, Berkowitz W F (1963). J Org Chem.

[37] Corey E J, Venkateswarlu A (1972). J Am Chem Soc.

[38] Kluge A F, Untch K G, Fried J H (1972). J Am Chem Soc.

[39] VanRheenen V, Kelly R C, Cha D Y (1976). Tetrahedron Lett.

[40] Sharpless K B, Akashi K (1976). J Am Chem Soc.

[41] Francais A, Bedel O, Haudrechy A (2008). Tetrahedron.

[42] Plattner J J, Gless R D, Rapoport H (1972). J Am Chem Soc.

[43] Mast C A, Eißler S, Stončius A, Stammler H-G, Neumann B, Sewald N (2005). Chem–Eur J.

[44] Wen S-J, Zhang H-W, Yao Z-J (2002). Tetrahedron Lett.

[45] Polniaszek R P, Belmont S E, Alvarez R (1990). J Org Chem.

[46] Butters M, Davies C D, Elliott M C, Hill-Cousins J, Kariuki B M, Ooi L-l, Wood J L, Wordingham S V (2009). Org Biomol Chem.

[47] Ukaji Y, Tsukamoto K, Nasada Y, Shimizu M, Fujisawa T (1993). Chem Lett.

[48] Pilli R A, Robello L G (2005). Synlett.

[49] Wang Y, Zhu S, Ma D (2011). Org Lett.

[50] Sakagami H, Kamikubo T, Ogasawara K (1996). Chem Commun.

