# Supplementary material for: A new approach toward the total synthesis of (+)-batzellaside B
Source: Beilstein J Org Chem. 2012 Oct 25;8:1831–8. doi: 10.3762/bjoc.8.210 (PMC3511019; doi:10.3762/bjoc.8.210)
Supplement: File 1 — Full experimental details and characterization data. [file Beilstein_J_Org_Chem-08-1831-s001.pdf]

Supporting Information

for

A new approach toward the total synthesis of (+)-batzellaside B

Jolanta Wierzejska, Shin-ichi Motogoe, Yuto Makino, Tetsuya Sengoku, Masaki Takahashi and Hidemi Yoda*

Address: Department of Materials Science, Faculty of Engineering, Shizuoka University, 3-5-1 Johoku, Naka-ku, Hamamatsu, Shizuoka 432-8561, Japan

Email: Hidemi Yoda - tchyoda@ipc.shizuoka.ac.jp

* Corresponding author

Full experimental details and characterization data

Table of contents

General methods S2
Synthesis and characterization of **9** S2
Synthesis and characterization of **10** S3
Synthesis and characterization of **11** S4
Synthesis and characterization of **6a** S5
Dihydroxylation of **6a** and characterization of **12a-A** and **12a–B** S5
Synthesis and characterization of **14B** S6
Synthesis and characterization of **6b–d** S8
General procedure for asymmetric dihydroxylation of **6a**–**d** S10
Synthesis and characterization of **12b–d** S11
Asymmetric dihydroxylation of **6d** S13
Synthesis and characterization of **13A** S13
Synthesis and characterization of **15** S15
Synthesis and characterization of **5** S16
Synthesis and characterization of **4** S17
General procedure for allylation of **4** S18

General: All solvents and reagents were of reagent-grade quality from Wako Pure Chemicals and Tokyo Chemical Industry (TCI) and used without further purification. The ^{1}H and ^{13}C nuclear magnetic resonance (NMR) spectra operating at the frequencies of 300 and 75 MHz, respectively, were recorded on a JEOL JNM-AL300 spectrometer in chloroform-*d* ($CDCl_3$) and methanol-d_4 (CD_3OD). Chemical shifts are reported in parts per million (ppm) relative to TMS, the solvent was used as the internal standard, and the coupling constants are reported in hertz (Hz). Fourier transform infrared (FTIR) spectra were recorded on a JASCO FT/IR-550 spectrometer. Melting points were measured with a Stanford Research Systems MPA100 automatic melting point apparatus. Elemental analyses were performed by JSL Model JM 10 instruments.

Synthesis and characterization of 9: To a solution of **8** (145 mg, 0.439 mmol) in methanol (0.50 mL) was added a solution of MeONa in methanol (1.0 M, 1.0 mL), and the resulting mixture was stirred at room temperature. After 10 min, the mixture was quenched by addition of saturated NH_4Cl_{aq} (10 mL) and concentrated in vacuo. The residue was extracted with ethyl acetate (20 mL). The extracts were washed with brine (30 mL), dried over anhydrous Na_2SO_4, filtered and concentrated in vacuo. The residue was purified by column chromatography on silica gel (eluent: hexane/ethyl acetate, 1/1) to yield **9** (156 mg, 0.430 mmol, 98%) as a colorless oil: IR ($CHCl_3$) 3450 cm^{-1} (N-H), 1741 cm^{-1} (C=O), 1718 cm^{-1} (C=O); ^{1}H NMR (300 MHz, $CDCl_3$) δ 4.60 (brs, 1H, N*H*), 3.56 (s, 3H, CH_3), 3.49 (brs, 3H, CH_2 and C*H*), 2.28 (t, *J* = 7.5 Hz, 2H, CH_2), 1.85-1.57 (m, 2H, CH_2), 1.32 (s, 9H, CH_3), 0.78 (s, 9H, CH_3), 0.06 (s, 6H, CH_3); ^{13}C NMR (75 MHz, $CDCl_3$) δ 174.1 (*C*), 155.8 (*C*), 79.0 (*C*), 65.1 (*C*H_2), 51.5 (*C*H_3), 30.7 (*C*H_2), 28.3 (*C*H_3), 27.0 (*C*H_2), 25.8 (*C*H_3), 18.2 (*C*), -5.6 (*C*H_3); Anal. calcd for $C_{17}H_{35}NO_5Si$: C, 56.47; H, 9.76; N, 3.87; found: C, 56.85; H, 9.39; N, 3.66.

Synthesis and characterization of 10: To a solution of **9** (43.9 mg, 0.122 mmol) in methanol (2.0 mL) was added a catalytic amount of *p*-toluenesulfonic acid (*p*-TsOH), and the resulting mixture was stirred at room temperature. After 3 h, the mixture was quenched by the addition of saturated $NaHCO_{3aq}$ (30 mL), concentrated, and extracted with ethyl acetate (50 mL). The extracts were washed with brine (30 mL), dried over anhydrous Na_2SO_4, filtered and concentrated in vacuo. The residue was roughly separated by column chromatography on silica gel (eluent: hexane/ethyl acetate, 1/1) to provide the crude alcohol intermediate (29.3 mg). To a solution of this material (29.3 mg) in acetone (2.5 mL) and 2,2-dimethoxypropane (DMP, 0.13 mL) was added boron trifluoride diethyl ether complex ($BF_3 \cdot Et_2O$, 1.3 mg, 0.089 mmol), and the resulting mixture was stirred at room temperature. After 30 min, the mixture was quenched by addition of saturated $NaHCO_{3aq}$ (10 mL), concentrated, and extracted with ethyl acetate (50 mL). The extracts were washed with brine (30 mL), dried over anhydrous Na_2SO_4, filtered and concentrated in vacuo. The residue was purified by column chromatography on silica gel (eluent: hexane/ethyl acetate, 2/1 to 1/1) to yield **10** (32.5 mg, 0.113 mmol, 93% for two steps) as a colorless oil: IR ($CHCl_3$) 1732 cm^{-1} (C=O), 1688 cm^{-1} (C=O); ^{1}H NMR (300 MHz, $CDCl_3$) δ 4.03-3.91 (m, 1H, CH_2), 3.90-3.80 (brs, 1H, C*H*), 3.72 (d, J = 7.8 Hz, 1H, CH_2), 3.68 (s, 3H, CH_3), 2.40-2.29 (m, 2H, CH_2), 2.13-1.76 (m, 2H, CH_2), 1.60 (s, 3H, CH_3), 1.56 (s, 3H, CH_3), 1.48 (s, 9H, CH_3); ^{13}C NMR (75 MHz, $CDCl_3$) δ 173.8 (*C*), 152.7 and 152.2 (rotamers, *C*), 94.1 and 93.7 (rotamers, *C*), 80.3 and 80.0 (rotamers, *C*), 67.1 (*C*H_2), 56.9 and 56.6 (rotamers, *C*H), 51.7 (*C*H_3), 30.9 (*C*H_2), 29.1 (*C*H_2), 28.5 (*C*H_3), 27.7 and 26.9 (rotamers, *C*H_3), 24.5 and 23.2 (rotamers, *C*H_3); Anal. calcd for $C_{14}H_{25}NO_5$: C, 58.52; H, 8.77; N, 4.87; found: C, 58.43; H, 8.51; N, 4.94.

Synthesis and characterization of 11: To a solution of diisopropylamine (1.56 g, 14.5 mmol) in THF (200 mL) was added a solution of *n*-butyl lithium (*n*-BuLi, 1.65 M, 8.5 mL, 14.0 mmol) in hexane at −78 °C, and the resulting mixture was stirred at this temperature. After 20 min, 1.4 M THF solution of **10** (2.02 g, 7.03 mmol) including hexamethylphosphoramide (HMPA, 3.36 g, 3.28 mmol) was added, and the resulting mixture was stirred. After 30 min, phenylselenyl bromide (PhSeBr, 2.48 g, 10.5 mmol) in THF (5 mL) was added to the reaction mixture, which was stirred at −78 °C. After 3 h, the mixture was quenched by the addition of saturated NH_4Cl_{aq} (20 mL), warmed to room temperature and extracted with ethyl acetate (100 mL). The extracts were washed with brine (50 mL), dried over anhydrous Na_2SO_4, filtered and concentrated in vacuo to provide the crude intermediate (3.94 g). To a solution of this material (3.94 g) in CH_2Cl_2 (140 mL) was added *m*-chloroperoxybenzoic acid (*m*-CPBA, 70%, 3.45 g, 14.0 mmol) at −40 °C. The resulting mixture was stirred for 3 h, quenched by the addition of saturated $NaHCO_{3aq}$ (30 mL), warmed to room temperature and extracted with CH_2Cl_2 (100 mL). The extracts were washed with brine (30 mL), dried over anhydrous Na_2SO_4, filtered and concentrated in vacuo. The residue was purified by column chromatography on silica gel (eluent: hexane/ethyl acetate, 8/1) to yield **11** (1.83 g, 6.41 mmol, 90% for two steps) as a colorless oil: IR ($CHCl_3$) 1714 cm^{-1} (C=O), 1693 cm^{-1} (C=O); ^1H NMR (300 MHz, $CDCl_3$) δ 6.85 (dd, *J* = 15.0 and 7.5 Hz, 1H, C*H*=C*H*), 5.93 (t, *J* = 15.0 Hz, 1H, C*H*=C*H*), 4.64-4.34 (brs, 1H, C*H*), 4.10 (dd, *J* = 9.0 and 6.3 Hz, 1H, CH_2), 3.80 (dd, *J* = 9.0 and 2.1 Hz, 1H, CH_2), 3.76 (s, 3H, CH_3), 1.80-1.33 (m, 15H, CH_3); ^{13}C NMR (75 MHz, $CDCl_3$) δ 166.9 (*C*), 152.3 and 151.9 (rotamers, *C*), 146.7 and 146.4 (rotamers, *C*H), 122.2 (*C*H), 94.8 and 94.3 (rotamers, *C*), 81.0 and 80.5 (rotamers, *C*), 67.5 (*C*H_2), 58.2 (*C*H_3), 51.8 (*C*H), 28.5 (*C*H_3), 27.7 and 26.6 (rotamers, *C*H_3), 24.8 and 23.7 (rotamers, *C*H_3); Anal. calcd for $C_{14}H_{23}NO_5$: C, 58.93; H, 8.12; N, 4.91; found: C, 58.64; H, 7.74; N, 4.80.

Synthesis and characterization of 6a: To a solution of **11** (533 mg, 1.87 mmol) in THF (5.1 mL) was added dropwise diisobutylaluminium hydride (DIBAL-H, 1.02 M in toluene, 4.2 mL, 4.3 mmol) at 0 °C. After 1 h, the mixture was poured into methanol (25 mL), followed by the addition of a saturated aqueous sodium potassium tartrate (10 mL), water (30 mL) and ethyl acetate (40 mL). The heterogeneous mixture was stirred overnight and extracted with ethyl acetate (50 mL). The extracts were washed with brine (30 mL), dried over anhydrous Na_2SO_4, filtered and concentrated in vacuo. The residue was purified by column chromatography on silica gel (eluent: hexane/ethyl acetate, 2/1) to yield **6a** (456 mg, 1.77 mmol, 95%) as a colorless oil: IR ($CHCl_3$) 3447 cm^{-1} (O-H), 1686 cm^{-1} (C=O); $[\alpha]_D^{30}$ +13 (*c* 0.98, $CHCl_3$); ^{1}H NMR (300 MHz, $CDCl_3$) δ 5.88-5.60 (m, 2H, C*H*=C*H*), 4.52-4.23 (brs, 1H, C*H*), 4.15 (d, *J* = 12 Hz, 2H, C*H*$_2$), 4.04 (dd, *J* = 9.0 and 6.3 Hz, 1H, C*H*$_2$), 3.74 (dd, *J* = 9.0 and 2.4 Hz, 1H, C*H*$_2$), 2.09 (brs, 1H, O*H*), 1.60 (s, 3H, C*H*$_3$), 1.51 (s, 3H, C*H*$_3$), 1.47 (s, 9H, C*H*$_3$); ^{13}C NMR (75 MHz, $CDCl_3$) δ 152.4 and 152.3 (rotamers, *C*), 131.4 (*C*H), 130.8 and 130.4 (rotamers, *C*H), 94.2 and 94.0 (rotamers, *C*) 80.5 and 79.9 (rotamers, *C*), 68.2 (*C*H$_2$), 63.0 (*C*H$_2$), 58.9 (*C*H), 28.6 (*C*H$_3$), 27.4 and 26.7 (rotamers, *C*H$_3$), 24.9 and 23.8 (rotamers, *C*H$_3$); Anal. calcd for $C_{13}H_{23}NO_4$: C, 60.68; H, 9.01; N, 5.44; found: C, 60.33; H, 8.90; N, 5.53.

Dihydroxylation of 6a and characterization of 12a-A and 12a–B: A mixture containing **6a** (52.0 mg, 0.202 mmol), 4-methylmorpholine-*N*-oxide (NMO, 0.13 mL, 0.606 mmol, 4.8 M aqueous solution) and osmium tetroxide (OsO_4, 0.81 mL, 0.0202 mmol, 0.025 M solution in *t*-BuOH) was stirred at room temperature. After 1 d, the reaction was quenched by addition of saturated $NaHSO_{3aq}$ (10 mL). The resulting mixture was extracted with ethyl acetate (3 x 30 mL). The extracts were dried over anhydrous Na_2SO_4, filtered and concentrated in vacuo. The residue was roughly

separated by column chromatography on silica gel (eluent: hexane/ethyl acetate, 3/1 to 0/1) to yield a 24:76 mixture of **12a-A** and **12a-B** (17.7 mg, 0.0607 mmol, 30%). These diastereoisomers were separated by the second column chromatography on silica gel (eluent: hexane/ethyl acetate, 1/2 then ethyl acetate/methanol, 1/1) to give pure triols **12a-A** and **12a-B** as white solids. **12a-A:** Mp 35–37 °C; IR (KBr) 3433 cm^{-1} (O-H), 3323 cm^{-1} (O-H), 1703 cm^{-1} (C=O); $[\alpha]_D^{30}$ +33 (*c* 1.0, $CHCl_3$); ^1H NMR (300 MHz, CD_3OD) δ 4.40-3.93 (brs, 3H, CH_2 and C*H*), 3.92-3.53 (brs, 4H, CH_2 and C*H*), 1.74-1.47 (m, 15H, CH_3); ^{13}C NMR (75 MHz, CD_3OD) δ 156.3 and 154.3 (rotamers, *C*), 95.5 (*C*), 82.5 and 81.6 (rotamers, *C*), 73.5 and 72.2 (rotamers, *C*H), 71.5 and 70.8 (rotamers, *C*H), 65.6 (CH_2), 64.7 (CH_2), 60.8 (*C*H), 28.8 (CH_3), 27.8 and 27.1 (rotamers, CH_3), 24.8 and 23.3 (rotamers, CH_3); Anal. calcd for $C_{13}H_{25}NO_6$: C, 53.59; H, 8.65; N, 4.81; found: C, 53.46; H, 8.39; N, 4.86. **12a-B**: Mp 117–121 °C; IR (KBr) 3406 cm^{-1} (O-H), 3317 cm^{-1} (O-H), 1658 cm^{-1} (C=O); $[\alpha]_D^{30}$ +14 (*c* 1.0, $CHCl_3$); ^1H NMR (300 MHz, CD_3OD) δ 4.37-4.12 (brs, 1H, C*H*), 4.11-3.83 (m, 2H, CH_2), 3.81-3.40 (m, 4H, CH_2 and C*H*), 1.68-1.25 (m, 15H, CH_3); ^{13}C NMR (75 MHz, CD_3OD) δ 156.0 (*C*), 95.3 (*C*), 82.8 (*C*), 72.2 (*C*H), 72.0 (*C*H), 66.4 (CH_2), 64.1 (CH_2), 60.4 and 60.3 (rotamers, *C*H), 28.8 (CH_3), 27.9 and 27.4 (rotamers, CH_3), 24.9 and 24.8 (rotamers, CH_3); Anal. calcd for $C_{13}H_{25}NO_6$: C, 53.59; H, 8.65; N, 4.81; found: C, 53.61; H, 8.29; N, 5.01.

Synthesis and characterization of 14B: To a solution of **12a-B** (92.7 mg, 0.318 mmol) in CH_2Cl_2 (0.60 mL) were added triethylamine (Et_3N, 96.5 mg, 0.954 mmol) and *tert*-butyldimethylsilyl chloride (TBSCl, 95.4 mg, 0.636 mmol) at 0 °C. The resulting mixture was stirred at room temperature for 1 d, cooled to 0 °C, quenched by the addition of water (10 mL), and extracted with ethyl acetate (30 mL). The extracts were washed with brine (20 mL), dried over anhydrous Na_2SO_4, filtered

and concentrated in vacuo. The residue was roughly separated by column chromatography on silica gel (eluent: hexane/ethyl acetate, 5/1) to yield crude TBS protected diol (128 mg). To a solution of this material (128 mg) in THF (2.5 mL) was added sodium hydride (NaH, 55% oil dispersion, 59.4 mg, 1.36 mmol) at 0 °C. After 20 min of stirring at this temperature, tetrabutylammonium iodide (Bu_4NI, 8.38 mg, 0.0227 mmol) and benzyl bromide (BnBr, 110 mg, 0.648 mmol) were added to the mixture. After being stirred at room temperature for 15 h, the mixture was quenched by the addition of saturated NH_4Cl_{aq} (10 mL), extracted with ethyl acetate (30 mL). The extracts were washed with brine (10 mL), dried over anhydrous Na_2SO_4, filtered and concentrated in vacuo. The residue was roughly separated by column chromatography on silica gel (eluent: hexane/ethyl acetate, 100/1 to 15/1) to yield crude **13B** (55.9 mg). To a solution of this material (78.7 mg) in methanol (4.5 mL) was added *p*-toluenesulfonic acid (*p*-TsOH, catalytic amount) and the resulting mixture was stirred at room temperature. After 2 d, the mixture was quenched by the addition of water (10 mL), concentrated, and extracted with ethyl acetate (30 mL). The extracts were washed with brine (20 mL), dried over anhydrous Na_2SO_4, filtered and concentrated in vacuo. The residue was roughly separated by column chromatography on silica gel (eluent: hexane/ethyl acetate, 1/1) to provide crude deprotected intermediate. To a solution of this material in CH_2Cl_2 (0.30 mL) was added trifluoroacetic acid (TFA, 0.18 mL), and the resulting mixture was stirred at room temperature for 30 min. Then, trifluoroacetic acid was removed azeotropically by using dioxane (1.0 mL) to provide the crude deprotected intermediate. To a solution of this material in a mixture of diethyl ether (1.0 mL) and water (1.0 mL) was added dropwise sodium periodate ($NaIO_4$, 34.0 mg, 0.16 mmol) at 0 °C. After being stirred at room temperature for 8 h, the mixture was extracted with CH_2Cl_2 (20 mL). The extracts were washed with brine (20 mL), dried over anhydrous Na_2SO_4, filtered

and concentrated in vacuo to provide crude lactol. To a solution of this material in CH_2Cl_2 (0.40 mL) were added powdered 4 Å molecular sieves (MS 4 Å, 139 mg), and the mixture was cooled to 0 °C. To this mixture, pyridinium chlorochromate (PCC, 33.7 mg, 0.156 mmol) was added, and the resulting mixture was warmed to room temperature with stirring for an additional 10 min. The resulting mixture was diluted with diethyl ether (5.0 mL), stirred for 5 h and filtered through a pad of Celite, which was washed successively with ethyl acetate (20 mL). The filtrate was concentrated in vacuo and the residue was purified by column chromatography on silica gel (eluent: hexane/ethyl acetate, 4/1 to 2/1) to yield **14B** (12.0 mg, 0.0402 mmol, 17% for 6 steps) as a colorless oil: IR ($CHCl_3$) 1793 cm^{-1} (C=O); $[\alpha]_D$ −51.6°(*c* 0.36, $CHCl_3$); ^1H NMR (300 MHz, $CDCl_3$) δ 7.40-7.25 (m, 10H, Ar*H*), 5.02 (d, *J* = 12.0 Hz, 1H, CH_2), 4.80 (d, *J* = 12.0 Hz, 1H, CH_2), 4.62 (d, *J* = 12.0 Hz, 1H, CH_2), 4.54 (d, *J* = 12.0 Hz, 1H, CH_2), 4.38 (dd, *J* = 21.0 and 15.0 Hz, 1H, C*H*), 4.31 (dd, *J* = 12.0 and 6.0 Hz, 1H, CH_2), 4.22 (d, 1H, *J* = 6.0 Hz, C*H*), 4.05 (dd, *J* = 9.0 and 6.0 Hz, 1H, CH_2); ^{13}C NMR (75 MHz, $CDCl_3$) δ 173.5 (*C*), 137.2 (*C*), 137.0 (*C*), 128.9 (*C*H), 128.8 (*C*H), 128.7 (*C*H), 128.6 (*C*H), 128.5 (*C*H), 128.2 (*C*H), 78.6 (*C*H), 77.6 (*C*H), 72.7 (*C*H_2), 72.5 (*C*H_2), 69.3 (*C*H_2); Anal. calcd for $C_{18}H_{18}O_4$: C, 72.47; H, 6.08; found: C, 72.13; H, 6.17.

Synthesis and characterization of 6b: To a solution of **6a** (506 mg, 1.97 mmol) in DMF (0.20 mL) were added imidazole (268 mg, 3.93 mmol) and TBSCl (445 mg, 2.95 mmol) at 0 °C. The resulting mixture was stirred at room temperature for 1 h, cooled to 0 °C, quenched by the addition of water (15 mL), and extracted with ethyl acetate (50 mL). The extracts were washed with brine (30 mL), dried over anhydrous Na_2SO_4, filtered and concentrated in vacuo. The residue was purified by column chromatography on silica gel (eluent: hexane/ethyl acetate, 5/1) to yield **6b** (562 mg,

1.51 mmol, 77%) as a colorless oil: IR ($CHCl_3$) 1687 cm^{-1} (C=O); $[\alpha]_D^{30}$ +19 (*c* 1.1, $CHCl_3$); ^1H NMR (300 MHz, $CDCl_3$) δ 5.85-5.55 (brs, 2H, C*H*=C*H*), 4.48-4.21 (brs, 1H, C*H*), 4.17 (s, 2H, CH_2), 4.01 (dd, *J* = 8.7 and 6.0 Hz, 1H, CH_2), 3.73 (dd, *J* = 8.7 and 2.1 Hz, 1H, CH_2), 1.59 (s, 3H, CH_3), 1.50 (s, 3H, CH_3), 1.46 (s, 9H, CH_3), 0.89 (s, 9H, CH_3), 0.05 (s, 6H, CH_3); ^{13}C NMR (75 MHz, $CDCl_3$) δ 152.3 (*C*), 131.6 (*C*H), 129.2 and 129.1 (rotamers, *C*H), 94.0 and 93.7 (rotamers, *C*), 80.1 and 79.7 (rotamers, *C*), 68.4 (*C*H_2), 63.2 (*C*H_2), 58.9 (*C*H), 28.6 (*C*H_3), 27.7 and 26.9 (rotamers, *C*H_3), 26.1 (*C*H_3), 24.7 and 23.9 (rotamers, *C*H_3), 18.5 (*C*), -5.1 (*C*H_3); Anal. calcd for $C_{19}H_{37}NO_4Si$: C, 61.41; H, 10.04; N, 3.77; found: C, 61.07; H, 9.65; N, 4.04.

Synthesis and characterization of 6c: To a solution of NaH (55% oil dispersion, 71.6 mg, 1.64 mmol) in THF (0.40 mL) was added **6a** (301 mg, 1.17 mmol) at 0 °C. After 20 min of stirring at this temperature, methyl chloromethyl ether (MOMCl, 132 mg, 1.64 mmol) was added to this reaction mixture. After being stirred at room temperature for 1 h, the mixture was quenched by the addition of saturated NH_4Cl_{aq} (10 mL), extracted with ethyl acetate (30 mL). The extracts were washed with brine (10 mL), dried over anhydrous Na_2SO_4, filtered and concentrated in vacuo. The residue was purified by column chromatography on silica gel (eluent: hexane/ethyl acetate, 5/1) to yield **6c** (241 mg, 0.800 mmol, 68%) as a colorless oil: IR ($CHCl_3$) 1689 cm^{-1} (C=O); $[\alpha]_D^{30}$ +24 (*c* 0.68, $CHCl_3$); ^1H NMR (300 MHz, $CDCl_3$) δ 5.88-5.65 (brs, 2H, C*H*=C*H*), 4.64 (s, 2H, CH_2), 4.53-4.20 (brs, 1H, C*H*), 4.19-3.97 (m, 3H, CH_2), 3.76 (dd, *J* = 8.7 and 1.5 Hz, 1H, CH_2), 3.37 (s, 3H, CH_3), 1.61 (s, 3H, CH_3), 1.51 (s, 3H, CH_3), 1.45 (s, 9H, CH_3); ^{13}C NMR (75 MHz, $CDCl_3$) δ 152.3 and 152.2 (rotamers, *C*), 132.4 and 132.1 (rotamers, *C*H), 128.3 (*C*H), 95.7 (*C*H_2), 94.2 and 93.9 (rotamers, *C*), 80.3 and 79.8 (rotamers, *C*), 68.3 (*C*H_2), 67.1 (*C*H_2), 58.9 (*C*H), 55.4 (*C*H_3), 28.5

(CH_3), 27.6 and 26.7 (rotamers, CH_3), 24.9 and 23.8 (rotamers, CH_3); Anal. calcd for $C_{15}H_{27}NO_5$: C, 59.78; H, 9.03; N, 4.65; found: C, 59.89; H, 9.42; N, 5.04.

Synthesis and characterization of 6d: To a solution of **6a** (373 mg, 1.45 mmol) in CH_2Cl_2 (14 mL) were added Et_3N (439 mg, 4.35 mmol), acetic anhydride (Ac_2O, 444 mg, 4.35 mmol) and 4-dimethylaminopyridine (DMAP, 177 mg, 1.45 mmol). The resulting mixture was stirred at room temperature for 1 h and concentrated. The resulting residue was poured into water (10 mL), extracted with ethyl acetate (30 mL). The extracts were dried over anhydrous Na_2SO_4, filtered and concentrated in vacuo. The residue was purified by column chromatography on silica gel (eluent: hexane/ethyl acetate, 6/1) to yield **6d** (434 mg, 1.44 mmol, 99%) as a colorless oil: IR ($CHCl_3$) 1734 cm^{-1} (C=O), 1691 cm^{-1} (C=O); $[\alpha]_D^{30}$ +11 (*c* 0.75, $CHCl_3$); ^1H NMR (300 MHz, $CDCl_3$) δ 5.85-5.60 (brs, 2H, C*H*=C*H*), 4.57 (m, 2H, CH_2), 4.49-4.19 (brs, 1H, C*H*), 4.05 (dd, *J* = 9.0 and 5.7 Hz, 1H, CH_2), 3.75 (dd, *J* = 9.0 and 2.1 Hz, 1H, CH_2), 2.07 (s, 3H, CH_3), 1.61 (s, 3H, CH_3), 1.51 (s, 3H, CH_3), 1.44 (s, 9H, CH_3); ^{13}C NMR (75 MHz, $CDCl_3$) δ 171.0 (*C*), 152.5 and 152.2 (rotamers, *C*), 134.0 and 133.5 (rotamers, *C*H), 126.2 (*C*H), 94.3 and 94.1 (rotamers, *C*), 80.5 and 80.0 (rotamers, *C*), 68.1 (CH_2), 64.4 (CH_2), 58.8 (*C*H), 28.5 (CH_3), 27.6 and 26.7 (rotamers, CH_3), 24.8 and 23.8 (rotamers, CH_3), 21.0 (CH_3); Anal. calcd for $C_{15}H_{25}NO_5$: C, 60.18; H, 8.42; N, 4.68; found: C, 60.36; H, 8.18; N, 4.71.

General procedure for asymmetric dihydroxylation of 6a–d (Table 1, entries 1–9): To a suspension of AD-mix-α or AD-mix-β in 50% aqueous *t*-BuOH (0.080 M, 0.50 mol %) were successively added a solution of olefinic substrate **6** in 50% aqueous *t*-BuOH (0.020 M) and methanesulfonamide (100 mol %) at 0 °C, and the resulting mixture was stirred at the temperature as indicated in Table 1. Upon

completion of the reaction based on TLC analysis, the mixture was quenched by addition of saturated $NaHSO_{3aq}$ (10 mL), and extracted with ethyl acetate (3 x 30 mL). The extracts were dried over anhydrous Na_2SO_4, filtered and concentrated in vacuo. The residue was roughly separated by column chromatography on silica gel to yield the mixtures of **12-A** and **12-B** (diastereomeric ratios and combined yields are presented in Table 1).

12b-A and 12b-B: Purification by column chromatography on silica gel (eluent: hexane/ethyl acetate, 3/1) gave mixtures of **12b-A** and **12b-B** as white solids (Table 1, entries 3,4), which were subjected to spectroscopic and elemental analyses without separation: Mp 92–94 °C; IR (KBr) 3446 cm^{-1} (O-H), 1656 cm^{-1} (C=O); ^{1}H NMR (300 MHz, $CDCl_3$) δ 4.60-4.18 (brs, 1H, C*H*), 4.17-4.10 (m, 1H, CH_2), 4.02-3.89 (m, 1H, CH_2), 3.84 (dd, *J* = 9.0 and 5.7 Hz, 1H, CH_2), 3.81-3.45 (m, 4H, CH_2 and C*H* and O*H*), 3.10-2.60 (brs, 1H, O*H*), 1.60 (s, 3H, CH_3), 1.52 (s, 3H, CH_3), 1.50 (s, 9H, CH_3), 0.90 (**12b-A**, s, 9H, CH_3), 0.89 (**12b-B**, s, 9H, CH_3), 0.08 (**12b-B**, s, 6H, CH_3), 0.07 (**12b-A**, s, 6H, CH_3); ^{13}C NMR (75 MHz, $CDCl_3$) δ 155.7 (**12b-A**, *C*), 154.7 (**12b-B**, *C*), 94.4 and 94.1 (rotamers, *C*), 81.7 (*C*), 73.3 (**12b-A**, *C*H), 71.6 (**12b-A**, *C*H), 71.4 (**12b-B**, *C*H), 69.6 (**12b-B**, *C*H), 66.0 (**12b-B**, *C*H), 65.0 (**12b-A**, *C*H), 64.8 (**12b-A**, *C*H_2), 63.8 (**12b-B**, *C*H_2), 59.7 (**12b-A**, *C*H_2), 59.2 (**12b-B**, *C*H_2), 28.5 (**12b-A**, *C*H_3), 28.5 (**12b-B**, *C*H_3), 27.6 and 27.2 (rotamers, *C*H_3), 26.0 (*C*H_3), 24.5 and 24.3 (rotamers, *C*H_3), 18.4 (**12b-A**, *C*), 18.3 (**12b-B**, *C*), -5.3 (**12b-B**, *C*H_3), -5.4 (**12b-A**, *C*H_3); Anal. calcd for $C_{19}H_{39}NO_6Si$: C, 56.26; H, 9.69; N, 3.45; found: C, 56.29; H, 9.38; N, 3.56.

12c-A and 12c-B: Purification by column chromatography on silica gel (eluent: hexane/ethyl acetate, 1/1) gave mixtures of **12c-A** and **12c-B** as colorless oils (Table

1, entries 5,6), which were subjected to spectroscopic and elemental analyses without separation: IR ($CHCl_3$) 3422 cm^{-1} (O-H), 1662 cm^{-1} (C=O); ^{1}H NMR (300 MHz, $CDCl_3$) δ 4.67 (s, 2H, CH_2), 4.51-4.24 (brs, 1H, C*H*), 4.23-4.10 (brs, 1H, CH), 4.07-3.75 (m, 2H, CH_2), 3.83-3.58 (m, 4H, C*H* and CH_2 and O*H*), 3.39 (**12c-A**, s, 3H, CH_3), 3.38 (**12c-B**, s, 3H, CH_3), 2.91-2.65 (brs, 1H, O*H*), 1.58-1.45 (m, 15H, CH_3); ^{13}C NMR (75 MHz, $CDCl_3$) δ 155.8 (**12c-A**, *C*), 154.9 (**12c-B**, *C*), 97.3 (**12c-B**, *C*H_2), 97.1 (**12c-A**, *C*H_2), 94.5 and 94.2 (rotamers, *C*), 82.1 and 81.9 (rotamers, *C*), 74.0 (**12c-A**, *C*H), 72.2 (**12c-B**, *C*H), 70.6 (**12c-A**, *C*H_2), 68.9 (**12c-B**, *C*H_2), 68.4 (*C*H), 66.0 (**12c-A**, *C*H_2), 65.0 (**12c-B**, *C*H_2), 59.8 (**12c-A**, CH), 59.2 (**12c-B**, CH), 55.6 (**12c-B**, *C*H_3), 55.5 (**12c-A**, *C*H_3), 28.5 (*C*H_3), 27.7 and 27.2 (rotamers, *C*H_3), 24.4 and 24.2 (rotamers, *C*H_3); Anal. calcd for $C_{15}H_{29}NO_7$: C, 53.72; H, 8.72; N, 4.18; found: C, 53.82; H, 8.35; N, 4.37.

12d-A and 12d-B: Purification by column chromatography on silica gel (eluent: hexane/ethyl acetate, 2/1) gave mixtures of **12d-A** and **12d-B** as white solids (Table 1, entries 7–9), which were subjected to spectroscopic and elemental analyses without separation: Mp 103–105 °C; IR (KBr) 3373 cm^{-1} (O-H), 3310 cm^{-1} (O-H), 1742 cm^{-1} (C=O), 1656 cm^{-1} (C=O); ^{1}H NMR (300 MHz, $CDCl_3$) δ 4.91-4.53 (brs, 1H, C*H*), 4.34-4.06 (m, 3H, CH_2 and C*H*), 3.98 (dd, *J* = 9.0 and 5.1 Hz, 1H, CH_2), 3.87 (dd, *J* = 9.0 and 5.1 Hz, 1H, CH_2), 3.82-3.68 (dd, *J* = 10.8 and 6.0 Hz, 1H, C*H*), 3.42 (t, *J* = 10.2 Hz, 1H, O*H*), 3.10-2.64 (brs, 1H, O*H*), 2.09 (**12d-A**, s, 3H, CH_3), 2.08 (**12d-B**, s, 3H, CH_3), 1.57-1.46 (m, 15H, CH_3); ^{13}C NMR (75 MHz, $CDCl_3$) δ 171.3 (**12d-A**, *C*), 171.2 (**12d-B**, *C*), 154.8 (*C*), 94.2 (*C*), 82.0 (*C*), 71.6 (*C*H), 67.5 (*C*H), 66.1 (**12d-A**, *C*H_2), 65.9 (**12d-B**, *C*H_2), 59.7 (**12d-A**, *C*H), 59.0 (**12d-B**, *C*H), 28.3 (*C*H_3), 27.7 (*C*H_3), 24.1 (*C*H_3), 20.9 (*C*H_3); Anal. calcd for $C_{15}H_{27}NO_7$: C, 54.04; H, 8.16; N, 4.20; found: C, 54.14; H, 7.94; N, 4.60.

Asymmetric dihydroxylation of 6d (Table 1, entry 10): To a suspension of AD-mix-α (294 mg, 0.50 mol %) in 50% aqueous *t*-BuOH (1.5 mL) was added a solution of **6d** (62.4 mg, 0.208 mmol) in 50% aqueous *t*-BuOH (0.50 mL) at 0 °C, and the resulting mixture was stirred at this temperature. After 3 d, the reaction was quenched by the addition of saturated $NaHSO_{3aq}$ (10 mL), and extracted with ethyl acetate (3 x 30 mL). The extracts were dried over anhydrous Na_2SO_4, filtered and concentrated in vacuo. The residue was roughly separated by column chromatography on silica gel (eluent: hexane/ethyl acetate, 2/1) to yield an 84:16 mixture of **12-A** and **12-B** (35.8 mg, 0.208 mmol, 52%).

Asymmetric dihydroxylation of 6d (Table 1, entry 11): To a suspension of AD-mix-α (133 mg, 0.50 mol%) in 50% aqueous *t*-BuOH (0.80 mL) were added a solution of **6d** (28.4 mg, 0.0948 mmol) in 50% aqueous *t*-BuOH (0.20 mL) and hydroquinine 1,4-phthalazinediyl diether ($(DHQ)_2PHAL$, 7.38 mg, 9.48 μmol) at 0 °C, and the resulting mixture was stirred at this temperature. After 3 d, the reaction was quenched by the addition of saturated $NaHSO_{3aq}$ (10 mL), and extracted with ethyl acetate (3 x 30 mL). The extracts were dried over anhydrous Na_2SO_4, filtered and concentrated in vacuo. The residue was roughly separated by column chromatography on silica gel (eluent: hexane/ethyl acetate, 2/1) to yield an 83:17 mixture of **12-A** and **12-B** (16.3 mg, 0.0497 mmol, 53%).

Synthesis and characterization of 13A: To a solution of a 84:16 mixture of **12d-A** and **12d-B** (752 mg, 2.26 mmol) in methanol (23 mL) was added potassium carbonate (K_2CO_3, 312 mg, 2.26 mmol), and the resulting mixture was stirred at room temperature for 1 h, quenched by the addition of saturated NH_4Cl_{aq} (10 mL) and concentrated. The residue was extracted with ethyl acetate (3 x 50 mL). The extracts

were dried over anhydrous Na_2SO_4, filtered and concentrated in vacuo. The residue was roughly separated by column chromatography on silica gel (eluent: hexane/ethyl acetate, 3/1 to 0/1) to yield an 84:16 mixture of **12a-A** and **12a-B** (654 mg). These diastereoisomers were separated by a second column chromatography on silica gel (eluent: hexane/ethyl acetate, 1/2 then ethyl acetate/methanol, 1/1) to give pure triols **12a-A** (548 mg) and **12a-B** (104 mg). To a solution of **12a-A** (548 mg) in CH_2Cl_2 (3.8 mL) were added Et_3N (761 mg, 7.52 mmol) and TBSCl (850 mg, 5.64 mmol) at 0 °C. The resulting mixture was stirred at room temperature for 1 d, cooled to 0 °C, quenched by the addition of water (10 mL), and extracted with ethyl acetate (30 mL). The extracts were washed with brine (20 mL), dried over anhydrous Na_2SO_4, filtered and concentrated in vacuo. The residue was roughly separated by column chromatography on silica gel (eluent: hexane/ethyl acetate, 5/1) to provide crude **12b-A** (671 mg). To a solution of this material (671 mg) in THF (17 mL) was added NaH (55% oil dispersion, 722 mg, 16.5 mmol) at 0 °C. After 20 min of stirring at this temperature, tetrabutylammonium iodide (Bu_4NI, 61.0 mg, 0.165 mmol) and BnBr (2.26 g, 13.2 mmol) were added to the mixture. After being stirred at room temperature for 3 d, the mixture was quenched by the addition of saturated NH_4Cl_{aq} (15 mL), and extracted with ethyl acetate (50 mL). The extracts were washed with brine (30 mL), dried over anhydrous Na_2SO_4, filtered and concentrated in vacuo. The residue was purified by column chromatography on silica gel (eluent: hexane/ethyl acetate, 100/1 to 15/1) to yield **13A** (657 mg, 1.12 mmol, 50% for 3 steps) as a white solid: Mp 51–53 °C; IR (KBr) 1687 cm^{-1} (C=O); $[\alpha]_D^{31}$ +20 (*c* 0.51, $CHCl_3$); ^1H NMR (300 MHz, $CDCl_3$) δ 7.40-7.15 (m, 10H, Ar*H*), 4.78-4.55 (m, 4H, PhCH_2), 4.47 (d, J = 8.4 Hz, C*H*), 4.35-4.05 (brs, 1H, C*H*), 3.90-3.76 (m, 2H, CH_2), 3.75-3.52 (m, 3H, CH and CH_2), 1.75-1.30 (m, 15H, CH_3), 0.86 (s, 9H, CH_3), 0.01 (s, 6H, CH_3); ^{13}C NMR (75 MHz, $CDCl_3$) δ 153.3 and 153.0 (rotamers, *C*), 139.2 (*C*), 139.0 (*C*), 128.5 (*C*H),

128.0 (*C*H), 127.7 (*C*H), 94.5 and 93.9 (rotamers, *C*), 80.4 and 80.2 (rotamers, *C*), 79.1 (*C*H), 77.4 and 76.7 (rotamers, *C*H), 73.9 (CH_2), 73.4 (CH_2), 62.7 (CH_2), 57.8 and 57.5 (rotamers, *C*H), 28.6 (CH_3), 27.6 and 27.0 (rotamers, CH_3), 26.0 (CH_3), 24.2 and 22.7 (rotamers, CH_3); Anal. calcd for $C_{33}H_{51}NO_6Si$: C, 67.65; H, 8.77; N, 2.39; found: C, 67.91; H, 8.73; N, 2.39.

Synthesis and characterization of 15: To a solution of **13A** (163 mg, 0.278 mmol) in THF (0.93 mL) was added tetra-*n*-butylammonium fluoride (TBAF, 1 M in THF, 0.42 mL, 0.416 mmol) at 0 °C. The resulting mixture was stirred at room temperature for 90 min, cooled to 0 °C, quenched by the addition of water (15 mL), and extracted with ethyl acetate (50 mL). The extracts were washed with brine (30 mL), dried over anhydrous Na_2SO_4, filtered and concentrated in vacuo. The residue was roughly separated by column chromatography on silica gel (eluent: hexane/ethyl acetate, 2/1) to provide the crude alcohol (128 mg). To a solution of this material (128 mg) in pyridine (0.13 mL) was added *p*-toluenesulfonyl chloride (*p*-TsCl, 155 mg, 0.813 mmol) at 0 °C. The resulting mixture was stirred at this temperature for 40 min, quenched by the addition of saturated NH_4Cl_{aq} (10 mL), and extracted with ethyl acetate (50 mL). The extracts were washed with brine (30 mL), dried over anhydrous Na_2SO_4, filtered and concentrated in vacuo. The residue was roughly separated by column chromatography on silica gel (eluent: hexane/ethyl acetate, 4/1) to provide the crude intermediate (165 mg). To a solution of this material (165 mg) in DMSO (2.6 mL) were added sodium cyanide (NaCN, 129 mg, 2.63 mmol) and $NaHCO_3$ (221 mg, 2.63 mmol) at room temperature. The resulting mixture was stirred at 60 °C for 8 h, cooled to room temperature, quenched by the addition of saturated NH_4Cl_{aq} (10 mL), and extracted with ethyl acetate (50 mL). The extracts were washed with brine (30 mL), dried over anhydrous Na_2SO_4, filtered and concentrated in vacuo. The

residue was purified by column chromatography on silica gel (eluent: toluene/ethyl acetate, 11/1) to yield **15** (106 mg, 0.221 mmol, 80% for 3 steps) as a colorless oil: IR ($CHCl_3$) 2250 cm^{-1} (C-N), 1692 cm^{-1} (C=O); $[\alpha]_D^{30}$ +30 (*c* 0.78, $CHCl_3$); ^{1}H NMR (300 MHz, $CDCl_3$) δ 7.45-7.23 (m, 10H, Ar*H*), 4.80-4.50 (m, 4H, PhCH_2), 4.45-4.15 (brs, 2H, C*H*), 4.15-3.67 (m, 3H, C*H* and CH_2), 2.78-2.45 (m, 2H, CH_2), 1.68 (s, 3H, CH_3), 1.60 (s, 3H, CH_3), 1.49 (s, 9H, CH_3); ^{13}C NMR (75 MHz, $CDCl_3$) δ 154.3 and 154.2 (rotamers, *C*), 138.1 (*C*), 137.5 (*C*), 128.8 (*C*H), 128.6 (*C*H), 128.5 (*C*H), 128.4 (*C*H), 128.4 (*C*H), 128.2 (*C*H), 118.3 (*C*), 94.4 and 94.2 (rotamers, *C*), 80.8 (*C*H), 78.7 and 78.2 (rotamers, *C*), 74.4 (*C*H), 74.1 (CH_2), 73.6 (CH_2), 64.8 (CH_2), 57.1 and 56.8 (rotamers, *C*H), 28.5 (CH_3), 27.4 and 27.0 (rotamers, CH_3), 24.1 and 22.4 (rotamers, CH_3), 20.4 (CH_2); Anal. calcd for $C_{28}H_{36}N_2O_5$: C, 69.98; H, 7.55; N, 5.83; found: C, 70.28; H, 7.36; N, 6.08.

Synthesis and characterization of 5: To a solution of **15** (38.7 mg, 0.0805 mmol) in methanol (2.7 mL) was added *p*-TsOH (20.8 mg, 0.121 mmol). The resulting mixture was stirred at room temperature for 6 h, quenched by the addition of saturated $NaHCO_{3aq}$ (10 mL), extracted with ethyl acetate (30 mL). The extracts were washed with brine (30 mL), dried over anhydrous Na_2SO_4, filtered and concentrated in vacuo. The residue was roughly separated by column chromatography on silica gel (eluent: hexane/ethyl acetate, 2/1) to yield crude alcohol intermediate (47.1 mg). To a solution of this material (250 mg, 0.567 mmol) in ethyl acetate (0.57 mL) were added silver oxide (Ag_2O, 657 mg, 2.84 mmol) and BnBr (243 mg, 1.42 mmol) and the resulting mixture was stirred at room temperature for 16 h. Then, the mixture was filtered through a pad of Celite, followed by successive washings with ethyl acetate (20 mL) and concentrated in vacuo. The residue was purified by column chromatography on silica gel (eluent: hexane/ethyl acetate, 3/1) to yield **5** (205 mg, 0.386 mmol, 67% for

2 steps) as a colorless oil: IR ($CHCl_3$) 3439 cm^{-1} (N-H), 2251 cm^{-1} (C-N), 1705 cm^{-1} (C=O); $[\alpha]_D^{30}$ +7.4 (*c* 1.0, $CHCl_3$); ^{1}H NMR (300 MHz, $CDCl_3$) δ 7.40-7.13 (m, 15H, Ar*H*), 4.86 (d, *J* = 9.1 Hz, 1H, N*H*), 4.75-4.34 (m, 6H, PhCH_2), 4.03 (dd, *J* = 15.0 and 9.3 Hz, 1H, C*H*), 3.92-3.71 (m, 2H, C*H*), 3.48-3.26 (m, 2H, CH_2), 2.80-2.50 (m, 2H, CH_2), 1.42 (s, 9H, CH_3); ^{13}C NMR (75 MHz, $CDCl_3$) δ 155.7 (*C*), 138.1 (*C*), 138.0 (*C*), 137.6 (*C*), 128.8 (*C*H), 128.7 (*C*H), 128.6 (*C*H), 128.4 (*C*H), 128.4 (*C*H), 128.3 (*C*H), 128.1 (*C*H), 118.1 (*C*), 80.2 (*C*), 77.7 (*C*H), 75.9 (*C*H), 75.1 (*C*H_2), 73.7 (*C*H_2), 73.2 (*C*H_2), 69.6 (*C*H_2), 49.3 (*C*H), 28.4 (*C*H_3), 20.1 (*C*H_2); Anal. calcd for $C_{32}H_{38}N_2O_5$: C, 72.43; H, 7.22; N, 5.28; found: C, 72.53; H, 7.60; N, 5.68.

Synthesis and characterization of 4: To a solution of **5** (36.0 mg, 0.0678 mmol) in toluene (0.9 mL) was added dropwise diisobutylaluminium hydride (DIBAL-H, 1.04 M in hexane, 0.098 mL, 0.102 mmol) at −78 °C. After 1 h with stirring at this temperature, the mixture was quenched by the addition of saturated aqueous sodium potassium tartrate (5 mL), warmed up to room temperature and poured into a mixture of water (3 mL) and ethyl acetate (5 mL). The heterogeneous mixture was stirred overnight and extracted with ethyl acetate (20 mL). The extracts were washed with brine (15 mL), dried over anhydrous Na_2SO_4, filtered and concentrated in vacuo. The residue was purified by column chromatography on silica gel (eluent: toluene/ethyl acetate, 6/1) to yield **4** (24.3 mg, 0.0455 mmol, 67%) as a colorless oil. Due to thermal instability attributed to equilibrium between cyclized and ring-opened species, the ^{13}C NMR spectrum of this material could not be obtained: IR ($CHCl_3$) 3503 cm^{-1} (O-H), 1694 cm^{-1} (C=O); ^{1}H NMR ($CDCl_3$) δ 7.31-7.26 (m, 15H, Ar*H*), 5.60 (brs, 1H, C*H*), 4.96-4.20 (m, 8H, PhCH_2 and C*H*), 3.99 (dd, *J* = 9.9 and 3.0 Hz, 1H, C*H*), 3.64-3.59 (m, 2H, CH_2), 2.26 (brs, 1H, O*H*), 1.65-1.43 (m, 11H, CH_3 and CH_2); Anal. calcd for $C_{32}H_{39}NO_6$: C, 72.02; H, 7.37; N, 2.62; found: C, 72.40; H, 7.62; N, 2.58.

General procedure for allylation of 4 (Table 2): An oven-dried round-bottom flask was purged with nitrogen and charged sequentially with a solution of **4** (0.1 M), allylic reagent and Lewis acid (solvents, temperatures, reagents and equiv are given in Table 2). The resulting mixtures were stirred until complete consumption of the starting material, quenched by slow addition of water (10 mL), warmed to room temperature, and extracted with ethyl acetate (50 mL). The extracts were washed with brine (30 mL), dried over Na_2SO_4, filtered and concentrated in vacuo. The residues were purified by column chromatography on silica gel (eluent: hexane/ethyl acetate, 30/1 to 15/1) to yield mixtures of **7** and **7'** as colorless oils (ratios of **7** and **7'** and combined yields are given in Table 2). These diastereoisomers could be separated by repeated column chromatography on silica gel: **7**: IR (NaCl) 1678 cm^{-1} (C=O); $[\alpha]_D^{30}$ +8.4 (*c* 1.2, $CHCl_3$); ^1H NMR (300 MHz, $CDCl_3$) δ 7.33-7.25 (m, 15H, Ar*H*), 5.69 (m, 1H, CH_2=C*H*), 4.95 (d, *J* = 9.6 Hz, 1H, CH_2=CH), 4.93 (d, *J* = 12.3 Hz, 1H, CH_2=CH), 4.90-4.43 (m, 6H, PhCH_2 and C*H*), 4.85 (m, 1H, C*H*), 4.18 (brs, 1H, C*H*), 3.88 (m, 1H, C*H*), 3.78 (dd, *J* = 10.5 and 4.5 Hz, 1H, CH_2), 3.61-3.57 (m, 2H, C*H*, CH_2), 2.36 (m, 1H, CH_2), 2.15 (m, 1H, CH_2), 1.99 (dd, *J* = 13.5 and 3.1 Hz, 1H, CH_2), 1.58-1.43 (m, 10H, CH_3 and CH_2); ^{13}C NMR (75 MHz, $CDCl_3$) δ 155.3 (*C*), 138.9 (*C*), 138.7 (*C*), 138.5 (*C*), 136.2 (*C*H), 128.4 (*C*H), 128.2 (CH), 127.7 (CH), 127.6 (CH), 127.5 (*C*H), 127.4 (*C*H), 116.9 (CH_2), 80.9 (*C*H), 80.0 (*C*), 73.0 (*C*H), 72.9 (CH_2), 72.9 (CH_2), 72.5 (CH_2), 69.7 (CH_2), 52.5 (*C*H), 50.4 (*C*H), 39.1 (CH_2), 31.9 (CH_2), 28.3 (CH_3); Anal. calcd for $C_{35}H_{43}NO_5$: C, 75.37; H, 7.77; N, 2.51; found: C, 75.66; H, 7.94; N, 2.90.

7': IR (NaCl) 1684 cm^{-1} (C=O); $[\alpha]_D^{24}$ +50 (*c* 1.1, $CHCl_3$); ^1H NMR (300 MHz, $CDCl_3$) δ 7.33-7.25 (m, 15H, Ar*H*), 5.76 (m, 1H, CH_2=C*H*), 5.07-5.01 (m, 2H, CH_2=CH), 4.67-4.49 (m, 6H, PhCH_2), 4.31 (brs, 1H, C*H*), 3.96 (t, *J* = 6.0 Hz, 1H, C*H*), 3.87-3.77 (m,

3H, C*H*, C*H*$_2$), 3.66 (dd, *J* = 9.9 and 3.9 Hz, 1H, C*H*$_2$), 2.55-2.32 (m, 2H, CH$_2$), 2.18 (dt, *J* = 14.8 and 6.0 Hz, 1H, C*H*$_2$), 1.87 (dt, *J* = 14.8 and 2.4 Hz, 1H, C*H*$_2$), 1.44 (s, 9H, C*H*$_3$); ^{13}C NMR (75 MHz, $CDCl_3$) δ 155.2 (*C*), 138.9 (*C*), 138.6 (*C*), 136.0 (*C*H), 128.3 (*C*H), 127.6 (*C*H), 127.5 (*C*H), 127.4 (*C*H), 117.2 (*C*H$_2$), 79.8 (*C*), 79.2 (*C*H), 76.8 (*C*H), 73.0 (*C*H$_2$), 72.2 (*C*H$_2$), 71.1 (*C*H$_2$), 68.5 (*C*H$_2$), 52.3 (*C*H), 51.0 (*C*H), 39.7 (*C*H$_2$), 28.4 (*C*H$_3$), 28.2 (*C*H$_2$); Anal. calcd for $C_{35}H_{43}NO_5$: C, 75.37; H, 7.77; N, 2.51; found: C, 75.38; H, 7.75; N, 2.84.
